# The role of 5-HT receptors in depression

**DOI:** 10.1186/s13041-017-0306-y

**Published:** 2017-06-24

**Authors:** Christine N. Yohn, Mark M. Gergues, Benjamin Adam Samuels

**Affiliations:** Department of Psychology, Behavioral & Systems Neuroscience Area, Rutgers, The State University of New Jersey, 152 Frelinghuysen Rd., Room 215, Piscataway, NJ 08816 USA

**Keywords:** Serotonin, Depression, Antidepressant, Dentate gyrus, 5-HT_1A_ receptor, Hippocampus, Adult neurogenesis

## Abstract

Depression is a polygenic and highly complex psychiatric disorder that remains a major burden on society. Antidepressants, such as selective serotonin reuptake inhibitors (SSRIs), are some of the most commonly prescribed drugs worldwide. In this review, we will discuss the evidence that links serotonin and serotonin receptors to the etiology of depression and the mechanisms underlying response to antidepressant treatment. We will then revisit the role of serotonin in three distinct hypotheses that have been proposed over the last several decades to explain the pathophysiology of depression: the monoamine, neurotrophic, and neurogenic hypotheses. Finally, we will discuss how recent studies into serotonin receptors have implicated specific neural circuitry in mediating the antidepressant response, with a focus being placed on the hippocampus.

## Introduction

Major depressive disorder (MDD) is a ubiquitous illness that plagues more than 300 million people worldwide across all races and socioeconomic groups [1, 2]. MDD often strikes early in life and remains a chronic or recurring lifelong illness, and is therefore responsible for more years lost to disability than any other illness [1]. Since MDD is characterized by diverse etiologies and an overlapping symptomology with highly comorbid disorders (i.e. anxiety), understanding the neurobiological basis of MDD is currently a major challenge for modern psychiatry and neurobiology [3, 4]. Overall, the underlying pathology of depression is extremely heterogenous and complex, which hinders the development of treatments that are effective for all depressed individuals.

Historically treatments have ranged from psychoanalysis and electroconvulsive therapy to modern medications such as antidepressants. The earliest drugs found to successfully treat depression were monoamine oxidase inhibitors (MAOIs). Iproniazid, the first MAOI, was actually developed to treat tuberculosis, but in the early 1950s it was found to elevate mood and stimulate patient activity [5]. MAOIs inhibit the oxidation of monoamines and ultimately result in increased extracellular levels of serotonin (5-HT), norepinephrine (NE), and dopamine (DA) throughout the brain. Tricyclics (TCAs), developed in the 1950s, were also found to be moderately effective antidepressants that increased monoamine levels mainly by blocking 5-HT and NE reuptake [6–8]. However, the acceptance and usage of these drugs were hindered by both pervasive public stigma and potentially severe side effects. By the late 1980s, second-generation antidepressants that were more pharmacologically specific, such as selective serotonin reuptake inhibitors (SSRIs), were developed and found to have improved side effect profiles. SSRIs inhibit 5-HT reuptake into raphe nuclei neurons, and chronic treatment results in increased 5-HT levels throughout the brain [9, 10]. The development of SSRIs resulted in adult use of antidepressants tripling between 1988 and 1994 and increasing an additional 48% from 1995 to 2002 [11]. Although developed several decades ago, SSRIs currently remain some of the most prescribed drugs in the world today.

The efficacy and actions of both first- and second-generation antidepressants are the principal basis of the monoamine hypothesis, which suggests that an imbalance in 5-HT, NE, and/or DA neurotransmission underlie the pathophysiology of depression [12, 13]. This hypothesis may also be supported by clinical observations dating back to the 1950s that reserpine, which depletes central stores of monoamines, can induce depression in a subset of patients [14, 15]. As for 5-HT specifically, acute tryptophan depletion induces the recurrence of mild depression symptoms in patients that demonstrated remission with 5-HT antidepressants [16–18]. Furthermore, cerebrospinal fluid levels of the primary metabolite of 5-HT (5-HIAA) appear to be lower in a subset of patients with MDD, especially those exhibiting suicidal behavior [19–21]. However, approximately 33% of MDD patients do not respond to treatment with a commonly used SSRI and 67% of patients do not remit to this first line treatment [22, 23]. Underscoring the diverse etiologies of MDD, in recent years some research has shifted focus to potential new therapies such as noncompetitive NMDA receptor antagonists [24, 25], anticholinergic agents [26], and opioid modulators [27–29]. Therefore, it will ultimately be critical to stratify patients into distinct subsets so that they can be treated with the most appropriate and effective medications.

This review addresses the roles that both different gene polymorphisms involved in 5-HT signaling and the different 5-HT receptors (i.e. 5-HT_1A_, 5-HT_1B_, 5-HT_4_, and 5-HT_7_) may have in the pathophysiology of depression and the antidepressant response. A streamlined knowledge of these 5-HT signaling-related polymorphisms and receptors may ultimately prove instructive in determining which patients will be responsive to SSRIs. Furthermore, the determination of specific spatial populations of 5-HT receptors involved in mediating the beneficial effects of antidepressant treatment will yield a window into the neural circuitry that modulates mood-related behaviors. Therefore, we will also discuss the location of the 5-HT receptors that mediate the antidepressant response and the neural circuitry that is directly affected by altered levels of 5-HT.

## SERT polymorphism

Within humans, variants that affect serotonergic function can affect disease susceptibility and response to antidepressant treatment. The most prominently studied polymorphism occurs in the promoter of the gene encoding the Serotonin Transporter (SERT), the protein that is the main target for many currently prescribed antidepressants. The promoter contains a polymorphism that results in a short (14 repeats) or long (16 repeats) allele. Individuals homozygous for the short SERT allele have decreased levels of SERT and enhanced susceptibility to stressful events and depression relative to individuals homozygous for the long SERT allele [30]. Additionally, aside from depression, the SERT polymorphism was originally proposed to account for 7–9% of the inherited variance in anxiety-related disorders [31]. However, recent genome-wide association data has found poor replication of candidate genes for MDD, including the SERT polymorphism [32]. In preclinical work, SERT deficiency is associated with increased anxiety and negative valence related behaviors in adulthood and a complete lack of responsiveness to SSRIs [33, 34].

## Serotonin receptors

### 5-HT_1A_

Accumulating evidence indicates a role for at least 5 of the 14 5-HT receptor subtypes: 5-HT_1A_, 5-HT_1B_, 5-HT_4_, 5-HT_6_, and 5-HT_7_. 5-HT_1A_ receptors (5-HT_1A_Rs) exist in two distinct populations: 1) as somatodendritic autoreceptors on the raphe nuclei neurons that produce 5-HT, and 2) as postsynaptic heteroreceptors that mediate local neuromodulatory effects in several brain areas innervated by serotonergic projections [35–38]. 5-HT_1A_Rs are Gi/o-coupled metabotropic receptors that, when activated, suppress cyclic adenosine monophosphate (cAMP) levels and ultimately inhibit neuronal activity [39]. Activation of 5-HT_1A_ autoreceptors decreases the firing rate of raphe nuclei neurons resulting in limited 5-HT release through a negative feedback mechanism [40]. 5HT_1A_ autoreceptors are associated with the etiology of anxiety behavior, as mouse studies suggest that specific modulation of 5-HT_1A_ autoreceptor levels on raphe nuclei neurons during discrete developmental windows can alter anxiety behavior in adulthood [38, 41–43].

In addition to SERT, a polymorphism also exists in the promoter region of the gene encoding the 5-HT_1A_R [44, 45]. This single nucleotide C(−1019) G polymorphism in the 5-HT_1A_R promoter alters binding of the transcriptional repressors NUDR/DEAF-1 and Hes5 such that repression is greatly reduced with the G(−1019) allele [46]. Presumably the lack of repression results in increased 5-HT_1A_R expression in the raphe nuclei of persons homozygous for the G(−1019 allele) and subsequently decreased serotonergic neuron firing. Indeed, preclinical work finds that mice deficient for the transcriptional repressor NUDR/DEAF-1 have upregulation of 5-HT_1A_ autoreceptors specifically in the raphe nuclei [47]. In humans, the G/G genotype is related to an increased risk of anxiety and MDD as well as a reduction in response to SSRI treatment [45, 48, 49].

With chronic SSRI treatment, the negative feedback mechanism that limits 5-HT release ultimately inactivates due to desensitization of the raphe 5-HT_1A_ autoreceptors and subsequent alterations in the firing rates of the serotonergic neurons, but this process can take weeks [40, 50, 51]. Through generation of transgenic mice, a preclinical study found that specifically altering levels of raphe 5-HT_1A_ autoreceptors could lead to the development of antidepressant responders and non-responders. Mice exhibiting lower levels of 5-HT_1A_ autoreceptors were more resilient to stress and more responsive to SSRI treatment than mice containing high levels of 5-HT_1A_ autoreceptors [38]. Importantly, the mice with the lower levels of 5-HT_1A_ autoreceptors also demonstrated a subchronic response to SSRIs in novelty suppressed feeding (NSF), a behavioral paradigm that usually requires chronic treatment of at least 14 days before an antidepressant response can be observed [38, 52]. Thus, raphe 5-HT_1A_ autoreceptors actually temporarily limit or inhibit the behavioral SSRI response due to their negative feedback on 5-HT release.

In addition to acting as an autoreceptor, 5-HT_1A_ is also a postsynaptic heteroreceptor that mediates responses to released 5-HT in several areas of the brain including the septum, hippocampus, amygdala, thalamus, and hypothalamus [53–55]. Several lines of evidence indicate a critical role for 5-HT_1A_ heteroreceptors in mediating the behavioral response to antidepressant treatment. Mice that are germline deficient (lacking both 5-HT_1A_ autoreceptors and heteroreceptors) do not respond to SSRIs in the NSF test, hinting at a potential role for the 5-HT_1A_heteroreceptors in mediating the behavioral response to antidepressants [56]. Additionally, chronic systemic treatment with the 5-HT_1A_R agonist 8-OH-DPAT mimics the behavioral effects of antidepressant treatment in the NSF test in wild-type but not 5-HT_1A_ receptor-deficient mice [56].

Chronic antidepressant treatment also results in increased adult hippocampal neurogenesis (discussed at length below), and this increase is necessary for the behavioral effects of antidepressants [56, 57]. Correlating with the behavioral effects, mice that are germline deficient for 5-HT_1A_ receptors do not show an increase in adult hippocampal neurogenesis with chronic SSRI treatment [56]. Furthermore, chronic treatment with the 5-HT_1A_R agonist 8-OH-DPAT also mimics the effects of antidepressants by increasing adult hippocampal neurogenesis [56].

In a recent study, Samuels and colleagues (2015) found that specific deletion of 5-HT_1A_ heteroreceptors from mature granule cells (GC) in the dentate gyrus (DG), a subfield of the hippocampus, abolished the effects of SSRIs in a variety of behavioral tasks (including NSF) and attenuated the effects of SSRIs on adult neurogenesis and hippocampal neurotrophic factor expression (BDNF and VEGF) [54]. By contrast, if 5-HT_1A_Rs were deleted from the young adult born granule cells (abGCs) in the DG, then the effects of SSRIs on behavior and neurogenesis remained intact. Furthermore, expressing 5-HT_1A_Rs in DG GCs on a 5-HT_1A_ deficient background demonstrated that this population of 5-HT_1A_Rs is sufficient to mediate the behavioral and neurogenic effects of SSRIs. Overall, the results from the series of experiments conducted by Samuels and colleagues (2015) indicate that dentate gyrus 5-HT_1A_ heteroreceptors on mature granule cells are a potential target for clinical therapeutics [54].

Previous clinical trials with drugs that target 5-HT_1A_Rs, such as pindolol, have yielded disappointing results likely because these drugs targeted both the autoreceptor and heteroreceptor populations, which can have somewhat opposing effects [58]. Future attempts at targeting 5-HT_1A_Rs should focus on specifically modulating the activity of either autoreceptors or heteroreceptors (but not both) in order to yield faster acting and/or improved antidepressants. To this end, recent pharmacological studies have reported a new generation of agonists that preferentially target 5-HT_1A_R subpopulations [53, 59].

### 5-HT_1B_

Levels of 5-HT_1B_Rs are also a key determinant of stress reactivity, and therefore 5-HT_1B_Rs may be a potential pharmacological target for antidepressant development [60, 61]. Unlike somatodendritic 5-HT_1A_ autoreceptors, 5-HT_1B_ G_αi_-coupled autoreceptors are located on both serotonergic and non-serotonergic presynaptic terminals throughout the brain where they inhibit neurotransmitter release [39, 50, 62–66]. Following the administration of SSRIs, mice lacking 5-HT_1B_ autoreceptors exhibit increases in 5-HT levels in the ventral hippocampus (vHPC) and decreases in anxiety-like behaviors [66]. Furthermore, chronic antidepressant treatment increases 5-HT release through decreasing the expression and efficacy of the 5-HT_1B_Rs in the dorsal raphe nuclei (DRN) [66–68]. However, data regarding whether 5-HT_1B_Rs facilitate the antidepressant response remain somewhat contradictory as some labs have found augmenting antidepressant effects of 5-HT_1B_Rs antagonists, while others have not [69–72]. Similar to the case with 5-HT_1A_Rs, the inconsistent pharmacological findings may be attributed to the dual function of 5-HT_1B_Rs as both heteroreceptors and autoreceptors. Additionally, due to the diffuse location of 5-HT_1B_ autoreceptors that overlap with 5-HT_1B_ heteroreceptors throughout the brain, it is difficult to delineate between the two distinct populations [63, 65, 66]. Similar to 5-HT_1A_ heteroreceptors, 5-HT_1B_ heteroreceptors on DG GCs may play a role in the SSRI-mediated increase in adult hippocampal neurogenesis [73, 74].

### 5-HT_2C_

5-HT_2C_Rs are G_αq_-coupled heteroreceptors that are expressed in several limbic structures including the hippocampus (especially enriched in CA3), amygdala, anterior olfactory and endopiriform nuclei, and cingulate and piriform cortex. Overactivity of 5-HT2CRs may contribute to the etiology of depression and anxiety as some suicide victims have abnormally high expression of 5-HT_2C_Rs in the prefrontal cortex [75]. Agomelatine, a mixed melatonergic agonist/5-HT_2C_R antagonist is an effective anxiolytic and antidepressant in both preclinical and clinical populations [76–80]. Furthermore, acute administration of SSRIs can lead to negative side effects (such as increased anxiety) presumably through activation of both 5-HT_1A_R autoreceptors and 5-HT_2C_R heteroreceptors [81–85].

Interestingly, a recent study from Marcinkiewcz et al. showed that 5-HT release from the dorsal raphe nucleus enhances fear and anxiety through activation of 5-HT_2C_Rs on a subpopulation of corticotropin-releasing factor (CRF) neurons in the bed nucleus of the stria terminalis (BNST) [86]. Ultimately, activation of these CRF neurons in the BNST engages an inhibitory microcircuit that silences outputs to the ventral tegmental area and lateral hypothalamus. Furthermore, Marcinkiewcz et al. demonstrated that acute SSRI treatment potentiates anxiety-like behavior and that this effect was blocked by specific chemogenetic inhibition of CRF neurons in the BNST [86]. Taken together, these results suggest that 5-HT_2C_Rs in the BNST underlie the negative effects of acute SSRI administration.

### 5-HT_4_

5-HT_4_Rs are G_αs_-coupled receptors that increase intracellular cAMP levels via adenylyl cyclase function to increase neuronal activity [39]. 5-HT_4_ heteroreceptors are widely expressed in limbic regions, including the amygdala, septum, and hippocampus as well as the mesolimbic system [39, 55].

The C-terminal tail of the 5-HT_4_R is subject to complex diversity due to alternative splicing of the mRNA resulting in several different variants [39]. Within this splice variant region are polymorphisms that are associated with susceptibility to unipolar depression [87]. In addition, a postmortem study revealed alterations in both 5-HT_4_R binding and cAMP concentration levels in several brain regions of depressed violent suicide victims [88]. One report also suggests that lower striatal 5-HT_4_R binding in humans may contribute to the etiology of MDD [89]. Together these results implicate a role for 5-HT_4_Rs in mood disorders.

5-HT_4_R expression is also associated with the development of some behavioral features of depression, since the deletion of or pharmacological blockade of 5-HT_4_Rs results in increased depressive and anxiety-like behaviors in rodents [74, 90, 91]. Interestingly, the 5-HT_4_R agonist (RS67333) produces rapid antidepressant effects after only three days of administration in rodents [92]. This short treatment window appears to be enough to both desensitize 5-HT_1A_ autoreceptors and increase hippocampal neurogenesis. A more recent study comparing RS67333 to fluoxetine (FLX) found that RS67333 induced anxiolytic-like effects in several behavioral tests after only 7 days, confirming that 5-HT_4_R agonists provide more rapid effects than currently used antidepressants [93]. Interestingly, administration of a 5-HT_4_R antagonists do not block the behavioral effects of SSRIs, indicating that 5-HT_4_R activation likely mediates anxiolytic-like effects via a distinct mechanism [94]. Thus, more research is needed to determine the therapeutic potential of 5-HT_4_Rs as a target for treating anxiety and depression.

### 5-HT_6_

5-HT_6_Rs are postsynaptic G_αs_-coupled heteroreceptors that are enriched in the striatum, nucleus accumbens (NAc), and cortex, with moderate expression in the hippocampus, amygdala, and hypothalamus [39]. A recent study found that two distinct agonists that are selective for 5-HT_6_Rs both produce antidepressant and anxiolytic-like effects in rodents [95]. Somewhat paradoxically, 5-HT_6_R antagonists also can induce antidepressant- and anxiolytic-like effects in rodent models [96–99]. It is currently unclear whether these similar behavioral outcomes are due to diverse neurochemical effects associated with 5-HT_6_R agonists and antagonists or whether distinct actions are being mediated in different brain regions [74, 98]. Future studies are necessary to further explore the role of 5-HT_6_R receptor subtypes in antidepressant-like responses.

### 5-HT_7_

5-HT_7_ are G_αs_-coupled heteroreceptors located in the limbic and cortical regions of the brain [39]. Hippocampal 5-HT_7_Rs appear to be involved in the interaction between the serotonergic system and the hypothalamus-pituitary-adrenal (HPA) axis since 5-HT_7_R agonists increase glucocorticoid receptor expression in hippocampal cell cultures [100]. Acute but not chronic, restraint stress increases 5-HT_7_R mRNA in hippocampal subregions CA2 and CA3 [101]. Antidepressant administration downregulates 5-HT_7_ in the hypothalamus [102]. Mice lacking 5-HT_7_Rs exhibit antidepressant-like behaviors in stressful environments and pharmacological blockade of 5-HT_7_Rs results in a faster antidepressant responses in rats [97, 103–107]. Furthermore, the atypical antipsychotic, amisulpride, also acts as an antidepressant that is a high affinity 5-HT_7_R antagonist. Interestingly, the antidepressant-like behavioral effects of amisulpride are abolished in mice lacking 5-HT_7_Rs [108]. Therefore, 5-HT_7_Rs antagonists may also represent a new class of antidepressants that could have faster therapeutic action in treating depression.

## Serotonin and Neurotrophic factors

Since the original development of the monoamine hypothesis of depression, more recent data has expanded this theory to the non-mutually exclusive neurotrophic and neurogenesis hypotheses. These hypotheses speculate that decreases in neurotrophic factors such as brain-derived neurotrophic factor (BDNF) or decreases in adult hippocampal neurogenesis are respectively involved in the pathophysiology of depression, and that their restoration is critical for the therapeutic efficacy of antidepressant treatment [109–113]. 5-HT signaling and 5-HT receptors are heavily involved in regulating the levels of both neurotrophic factors and adult hippocampal neurogenesis.

The neurotrophic hypothesis is supported by the idea that stress and/or depression decrease expression of various neurotrophic factors (i.e. BDNF) in limbic areas and this decrease correlates with neuronal atrophy [110, 111, 114]. Specifically, following exposure to stressful experiences researchers have observed decreases in BDNF in rodent hippocampus and prefrontal cortex [109, 111, 115, 116]. Similarly, in humans, postmortem studies find reduced levels of BDNF in these regions of depressed patients [111, 117, 118]. In both humans and rodents, chronic SSRI treatment increases BDNF levels [111, 119, 120] with BDNF signaling required for adult hippocampal neurogenesis, synaptic plasticity, and neuronal remodeling [121, 122]. In mice lacking BDNF in the forebrain or the BDNF receptor Tropomysin receptor kinase B (TrkB) in adult DG neural precursor cells (NPCs), the behavioral and adult neurogenic response to SSRI treatment is eliminated [121, 123]. SSRI administration increases the maturation of young abGCs, as measured by dendritic arborization complexity [124]. BDNF and activation of its receptor TrkB have similar effects on maturation of young adult born neurons, suggesting that BDNF may mediate some of the effects of SSRIs on neurogenesis [125–128]. Interestingly, direct infusions of BDNF into the DG of rodents results in antidepressant-like behavioral effects [129].

In addition to BDNF, other neurotrophic factors such as vascular endothelial growth factor (VEGF), fibroblast growth factor 2 (FGF2), insulin-like growth factor 1 (IGF1), and Activin-A are also increased by antidepressant treatment. Unlike the established association between BDNF levels and adult hippocampal neurogenesis, these neurotrophic factors are implicated to varying degrees in mediating effects on neurogenesis and synaptogenesis [127, 130–136]. Similar to BDNF, direct cerebral infusions of any one of these growth factors can result in antidepressant-like behavioral responses [127, 129, 130, 134, 136, 137].

In humans, a common single nucleotide polymorphism (SNP) that results in a methionine substitution for valine at codon 66 (Val66Met) in the 5′ pro-domain of the BDNF coding region occurs in 25–32% of the Caucasian population and in 40–50% of the Asian population [138–140]. In the Caucasian population, the Val/Val allele is associated with higher neurotic scores and higher levels of trait anxiety than subjects with the Val/Met or Met/Met genotypes. By contrast, in Asian populations, the Met/Met allele is associated with expression of suicidal and psychotic symptoms and depression in the elderly [141, 142]. Chen and colleagues (2006) recreated this SNP in mice and observed that the BDNF variant (Met/Met) mice had increased anxiety related behaviors when placed in a stressful environment [143]. Furthermore, antidepressants were ineffective in treating this increased anxiety [143].

Some recent studies suggest that there may be epistatic interactions between the C(−1019)G polymorphism in the promoter of the gene encoding 5-HT_1A_R and other gene polymorphisms such as the SNP found in the gene encoding BDNF [144–146]. As an example, subjects with both the G/G genotype in the 5-HT_1A_R promoter and at least one copy of the Met allele of the BDNF Val66Met polymorphism had a greater than three times higher risk of treatment resistant depression [144].

Several studies attempt to directly link the role of BDNF and other neurotrophic factors with 5-HT receptors and signaling [54, 147–149]. For instance, in vitro studies show that BDNF dose-dependently decreases 5-HT reuptake, suggesting a direct effect on the function of SERT [150]. Since expression of BDNF and other neurotrophic factors are positively regulated by activity, activation of 5-HT receptors positively coupled to cAMP levels (such as 5-HT_4_ and 5-HT_7_) should yield enhancement of neurotrophic factor levels. The 5-HT_4_R agonist RS67333 increases BDNF mRNA expression in the hippocampus [151]. Furthermore, in vitro studies show that the 5-HT_7_R agonist LP12 increases expression of the BDNF receptor TrkB [152]. By contrast, specific deletion of 5-HT_1A_Rs, which are negatively coupled to cAMP levels, from mature DG GCs attenuates the chronic SSRI-induced increase in BDNF and VEGF levels [54]. While there is precedent for 5-HT_1A_R mediated regulation of VEGF levels in the dentate gyrus, this data is surprising given that 5-HT_1A_Rs receptors are inhibitory and both BDNF and VEGF activity are induced by activity [39, 153]. However, since findings from Samuels and colleagues (2015) are based on chronic, not acute, SSRI administration, it is possible that the effects are mediated through an indirect downstream mechanism that has yet to be resolved [54].

The FGF receptor FGFR1 can form heteroreceptor complexes with 5-HT_1A_Rs in the hippocampus and raphe nucleus [131, 154, 155]. Treatment with 5-HT_1A_R agonists or SSRIs results in activation of FGFR1 signaling [131, 156]. Additionally, transactivation of these receptor complexes results in synergistic increases in neurite density and protrusions, suggesting a combined role of FGFR1 and 5-HT_1A_Rs in synaptogenesis [156]. Furthermore, formation of FGFR1–5-HT_1A_R heterocomplexes may cause uncoupling of GIRK-5-HT_1A_R heterocomplexes in the raphe nuclei [154]. Theoretically would decrease 5-HT_1A_R autoreceptor function, so direct targeting of FGFR1–5-HT_1A_R heterocomplexes could result in faster acting antidepressants. Overall, 5-HT receptors and neurotrophic factors appear to be synergistically involved in both the pathophysiology of depression and the antidepressant response.

## Serotonin and Neurogenesis

Over the last two decades, it has become accepted that new neurons are produced in mammals in two discrete locations, the subventricular zone (SVZ) of the lateral ventricle and the subgranular zone (SGZ) of the DG in the hippocampus [157]. The neurons born in the SVZ migrate through the rostral migratory stream into the olfactory bulb and become interneurons, while those born in the SGZ migrate into the granular layer of the DG and eventually develop into mature granule neurons. The process of adult neurogenesis involves several steps, which include proliferation and fate specification of neural progenitors, neuronal migration and maturation, as well as synaptic integration of young neurons into the existing neuronal circuitry. Various well-established molecular markers are used to identify cells at distinct points, with electrophysiological cell membrane properties well understood throughout the neurogenesis process [157, 158].

Chronic, but not acute, antidepressant treatment increases proliferation of dividing NPCs in the SGZ, differentiation of precursor cells into young abGCs, and the rate by which young abGCs mature and integrate into the DG circuitry [57, 124]. Furthermore, the effects of chronic antidepressants seem to be specific to the SGZ as they do not increase neurogenesis in the SVZ [57, 159]. Critically, ablation of the adult hippocampal neurogenic niche, by focal radiological approaches, results in a loss of the behavioral antidepressant response, suggesting a necessary role for adult neurogenesis in mediating the behavioral effects of chronic antidepressant treatment [56, 160, 161]. These studies directly resulted in the neurogenesis hypothesis [112, 113]. However, it is important to note that ablation of adult hippocampal neurogenesis in rodents does not result in increases in anxiety- and depression-related behaviors [56, 161]. Similarly, while decreases in the number of DG GCs have been found in postmortem samples of untreated depressed patients, there does not appear to be a decrease in the number of progenitor cells [162]. Furthermore, specifically enhancing neurogenesis via a genetic approach does not result in an antidepressant-like phenotype under baseline conditions [163]. Therefore, while increasing adult hippocampal neurogenesis is necessary for the antidepressant response, it is not sufficient to mediate an antidepressant response and there is limited data to suggest that decreases in adult hippocampal neurogenesis may underlie the pathophysiology of depression.

The mechanisms by which SSRIs increase adult hippocampal neurogenesis is likely mediated by several different 5-HT receptors. Administration of the 5HT_1A_R/5-HT_7_R agonist 8-OH-DPAT increases neurogenesis in both the SGZ and SVZ [56, 73]. Furthermore, SSRIs do not increase neurogenesis in mice that are germline deficient for 5-HT_1A_Rs [56]. Interestingly, the recent study by Samuels and colleagues demonstrated that specific deletion of 5-HT_1A_Rs from mature DG GCs, but not from young abGCs, abolished the behavioral response to SSRI treatment and attenuated the neurogenic response [54]. Taken together, these data indicate that 5-HT_1A_Rs are likely a major target for SSRI-induced increases in adult hippocampal neurogenesis.

Similar to 5HT_1A_Rs, 5-HT_4_Rs appear to be associated with adult neurogenesis, since 5-HT_4_R agonists increase neurogenesis in the DG and in the enteric nervous system [92, 93, 151, 164–166]. By contrast, 5-HT_4_R antagonists reduce differentiation of NPCs with minimal effect on cell proliferation, maturation, or morphology [93, 164]. Furthermore, the beneficial effects of 5-HT_4_R agonists are not only rapid acting on behavior but also on adult hippocampal neurogenesis. Three days of treatment with the 5-HT_4_R agonist RS67333 significantly increases adult hippocampal neurogenesis [92, 151]. However, recent data suggests that the rapid behavioral effects of 5-HT_4_R agonists are mediated by a neurogenesis-independent mechanism [93]. Importantly, similar to 5-HT_1A_R, mice that are 5-HT_4_R germline deficient also show an attenuated neurogenic response to chronic SSRI treatment [167].

One interesting alternative to the traditional neurogenesis hypothesis is that SSRI treatment may also cause mature GCs in the DG to undergo a dematuration process that yields cells with properties more similar to young abGCs. Chronic SSRI treatment causes a decrease in expression of the mature granule cell marker calbindin in the DG [167, 168]. Therefore, it is possible that what is commonly measured to be maturation of young adult born granule cells (assessed by dendritic complexity of Dcx-positive cells) could also be dematuration of previously mature granule cells. Furthermore, this dematuration phenomenon is attenuated in mice germline deficient for the 5-HT_4_R [168]. Further studies have found that chronic SSRI treatment can also induce dematuration of parvalbumin-positive interneurons in the basolateral amygdala and the frontal cortex in adult mice [169, 170]. Thus, the antidepressant response may rely on both increases in neurogenesis and dematuration. It would be particularly interesting to determine whether signaling via distinct serotonin receptors can result in either increases in neurogenesis or dematuration. Further work using both spatially restricted 5-HT_1A_R and 5-HT_4_R deficient mice is required to further address this hypothesis.

In addition, while not nearly as well established as SGZ and SVZ adult neurogenesis, several studies have suggested that adult neurogenesis can occur in other brain regions such as the cortex and hypothalamus [171–173]. A recent study by Ohira and colleagues (2013) found that SSRI treatment increased cortical inhibitory neuron proliferation [173]. Some have speculated that GABAergic interneurons are involved in the etiology of depression [174], so it will be interesting to determine whether cortical neurogenesis plays a role in mediating the beneficial effects of antidepressants on behavior.

## Serotonin and the neural Circuity of the hippocampus

**Fig. 1:**
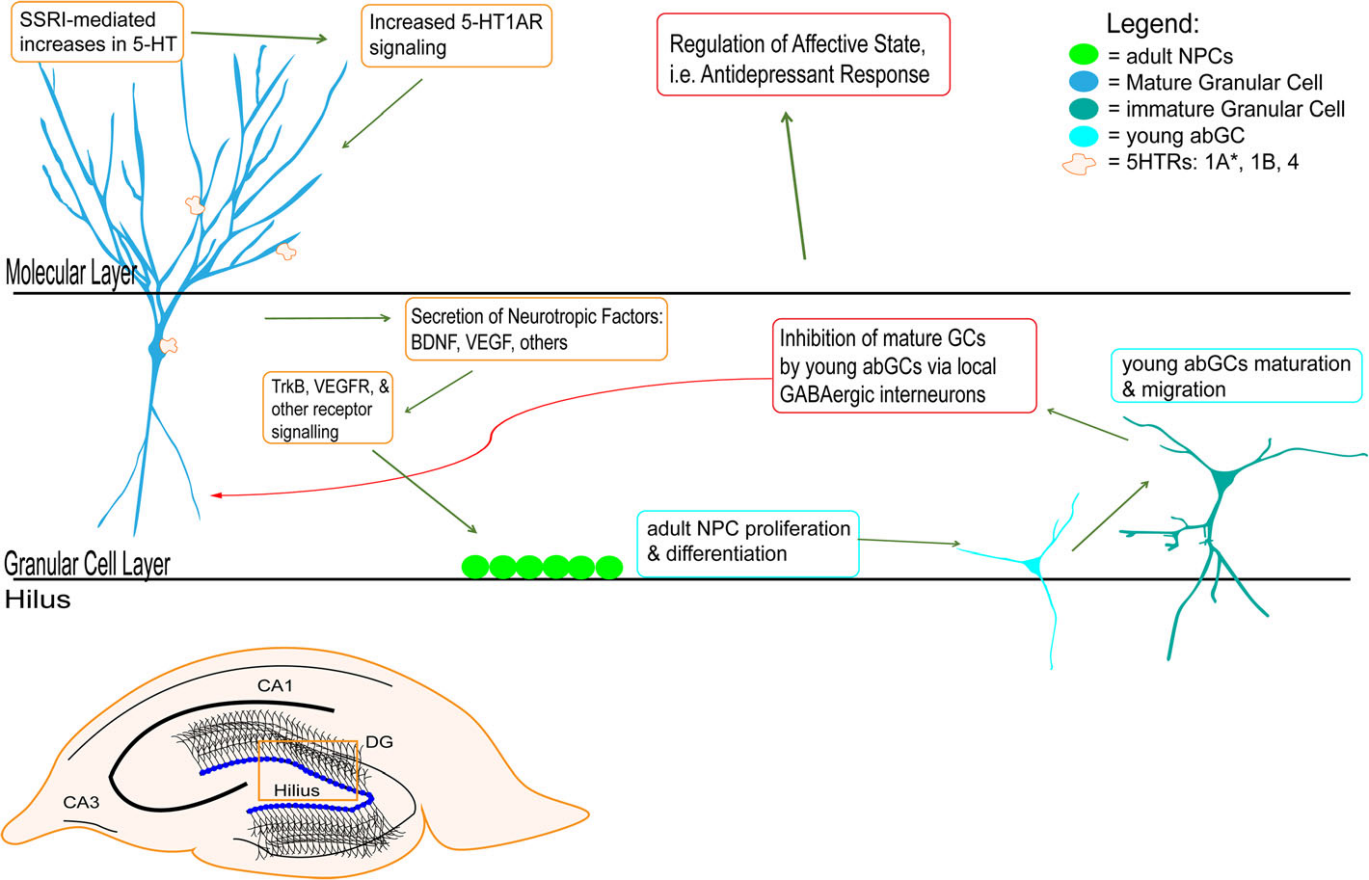
A proposed model of the hippocampal microcircuit underlying the effects of increased serotonin on the dentate gyrus. First, chronic SSRI administration increases 5-HT levels, which results in activation of 5-HTRs on dentate gyrus granule cells. Activation of 5HT-_1A_Rs on mature granular cells ultimately results in release of downstream growth factors such as BDNF, VEGF, and others, which bind to receptors on neural precursor cells (NPCs) in the subgranular zone. NPCs then proliferate and differentiate into young adult born granule cells (abGCs), which will begin to migrate, mature, and finally integrate into the granule cell layer. However, the young abGCs have distinct plasticity properties from the mature dentate gyrus granule cells and activate local GABAergic interneurons to evoke strong inhibitory input to the mature granule cells

The results from Samuels and colleagues (2015) suggest that 5-HT_1A_Rs on mature DG GCs are critical mediators of the effects of SSRIs on behavior, neurotrophic factors, and neurogenesis [54]. We propose that chronic activation of 5-HT_1A_Rs on mature DG GCs activate signaling cascades that ultimately result in secretion of neurotrophic factors, such as BDNF and VEGF, which in turn stimulate proliferation of NPCs as well as differentiation and maturation of young abGCs (Fig. 1). The young abGCs, which have distinct plasticity properties from the mature DG GCs, can then activate local GABAergic interneurons to evoke strong inhibitory input to the mature GCs [175–178] (Fig. 1). In this model inhibition of mature GCs via direct activation of 5-HT_1A_Rs or via the local microcircuitry is therefore critical for the antidepressant response.

Interestingly, 5-HT_1A_Rs show a unique expression pattern in the rodent DG as expression levels dramatically increase along the dorsoventral axis to the point that the vast majority of DG 5-HT_1A_Rs are expressed in the ventral pole [55]. Several studies imply that the dorsal hippocampus (dHPC) and vHPC may serve different functions, where the dHPC is more involved in cognitive functions, while the vHPC is important in regulating emotional affective states [179]. For instance, dHPC lesions reduce spatial memory in Morris water maze and radial arm maze whereas by vHPC lesions do not impair spatial memory [180, 181]. More modern approaches demonstrate that specific optical stimulation (via channel rhodopsin 2, ChR2) of basolateral amygdala (BLA) to vHPC projections or vHPC projections to NAc increases anxiety-related behaviors [182, 183]. By contrast, inhibition of vHPC projections to medial prefrontal cortex (mPFC) decreases anxiety-related behavior [184]. Directly activating granule neurons in the dorsal DG with acute stimulation of ChR2 reduces freezing behavior and recall in the contextual fear conditioning paradigm, however, this effect is not seen when stimulating the vHPC [181]. Furthermore, acute optogenetic inhibition (using halorhodopsin) of dorsal DG, but not ventral DG, leads to reductions in freezing behavior when photoillumination occurs during encoding and mice are tested 24 h later. By contrast, acute optogenetic inhibition of ventral DG but not dorsal DG results in anxiolytic-like behavioral effects.

**Fig. 2:**
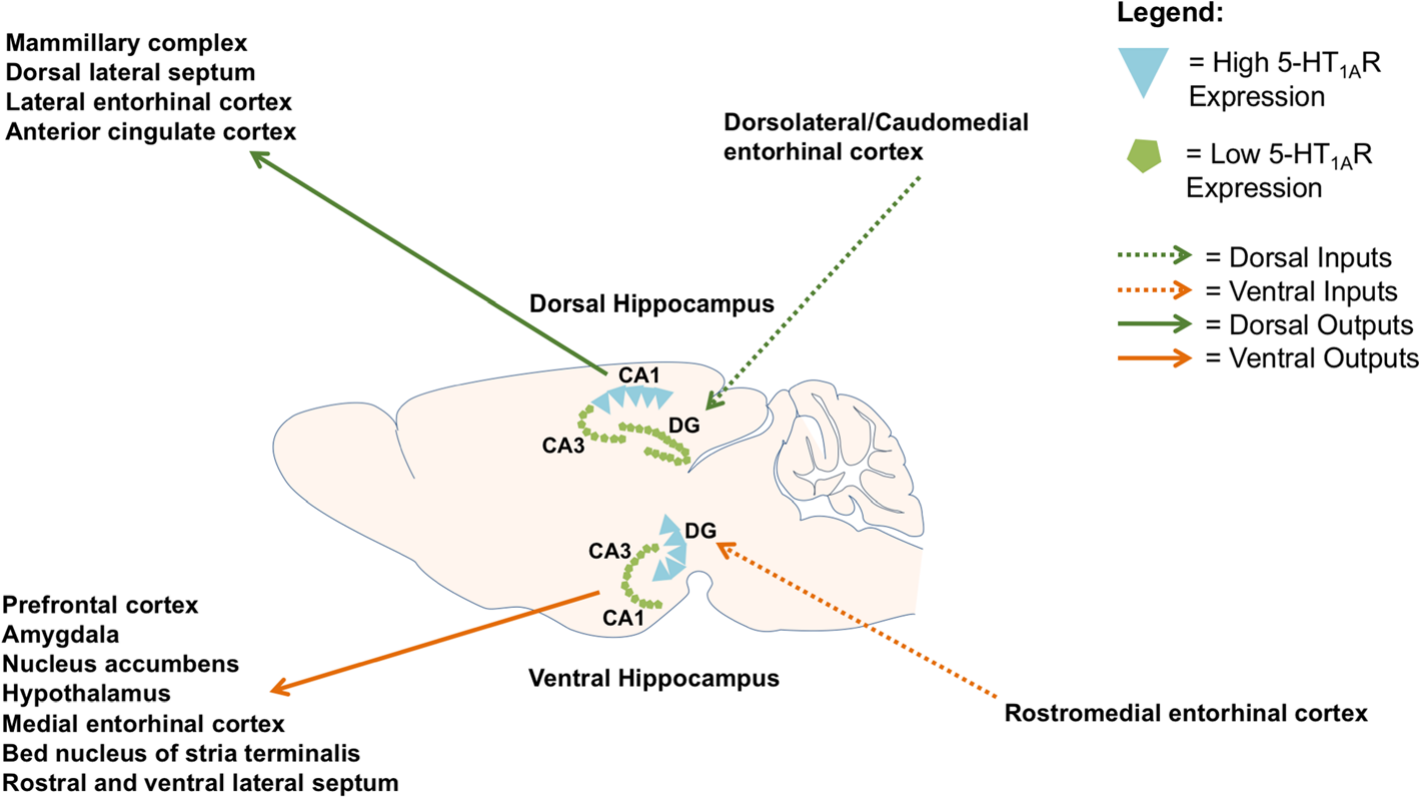
The expression of 5-HT_1A_ receptors along the dorsoventral axis of the hippocampus in a rodent brain. 5-HT_1A_R expression is highest in dorsal CA1 and ventral dentate gyrus. The dorsal and ventral hippocampus participate in distinct circuitry, with the ventral hippocampus projecting to limbic structures. Therefore, 5-HT_1A_Rs on dentate gyrus granule cells are well positioned to exert an influence on mood related behaviors

The different roles dorsal and ventral DG have in mediating diverse behaviors is likely due to a distinct connectivity. Dorsal DG receives inputs from dorsolateral and caudomedial entorhinal cortex, and medial septal nucleus, which relay inputs from V1, S1, and thalamic nuclei. Efferent outputs from dorsal DG are sent to the mammillary complex, dorsal lateral septum, lateral entorhinal cortex, and anterior cingulate cortex [179, 185] (Fig. 2). Many of these regions are critical for memory, locomotion, and exploration, thereby demonstrating the importance of the dHPC in cognitive rather than mood related tasks. Conversely, the ventral DG receives inputs from rostromedial entorhinal cortex and medial septal nucleus that convey information from auditory and piriform cortices. Unlike dorsal DG, ventral DG projects to areas important for regulating emotional affect, with outputs extending to the prefrontal cortex, NAc, hypothalamus, amygdala, medial entorhinal cortex, BNST, as well as rostral and ventral lateral septal nuclei (Fig. 2) [179, 185].

Aside from circuit connectivity, there are electrophysiological, molecular, and anatomic differences between the dHPC and vHPC [179]. The vHPC has higher levels of 5-HT and 5-HT innervation relative to the dHPC, demonstrating the importance of 5-HTR signaling within the vHPC in potentially mediating emotional affect and antidepressant response [186]. In the hippocampus, 5-HT_1A_Rs are highly expressed in the ventral DG and dorsal CA1, two distinct hippocampal subfields [55] (Fig. 2). Given that dentate gyrus 5-HT_1A_Rs are necessary and sufficient for mediating the behavioral effects of SSRIs, their location in the ventral pole positions these receptors to directly influence limbic circuitry in order to regulate mood-related behavior. Future work is necessary to determine whether specific pharmacological or electrical manipulations of ventral DG may be a novel therapeutic avenue for the treatment of depression and anxiety.

## Abbreviations

5-HT: Serotonin
5-HT_1A_: Serotonin receptor 1A
5-HT_1B_: Serotonin receptor 1B
5-HT_2C_: Serotonin receptor 2C
5-HT_4_: Serotonin receptor 4
5-HT_6_: Serotonin receptor 6
5-HT_7_: Serotonin receptor 7
abGCs: Adult born granule cells
BDNF: Brain-derived neurotrophic factor
cAMP: Cyclic adenosine monophosphate
ChR2: Channelrhodopsin 2
DA: Dopamine
DG: Dentate gyrus
DHPC: Dorsal hippocampus
DRN: Dorsal raphe nucleus
FGF: Fibroblast growth factor
FGFR: Fibroblast growth factor receptor
GC: Granule cells
GCL: Granular cell layer
HPA: Hypothalamic-pituitary-adrenal
IGF: Insulin-like growth factor
MAOIs: Monoamine oxidase inhibitors
MDD: Major Depressive Disorder
NE: Norepinephrine
NPC: Neural progenitor cells
NSF: Novelty suppressed feeding
SERT: Serotonin transporter
SGZ: Subgranular zone
SSRIs: Selective serotonin reuptake inhibitors
SVZ: Subventricular zone
TCAs: Tricyclic antidepressants
VEGF: Vascular endothelial growth factor
VEGFR: Vascular endothelial growth factor receptor
VHPC: Ventral hippocampus

## References

[1] Smith K (2014). Mental health: a world of depression. Nature.

[2] Murray CJ, Lopez AD (1996). Evidence-based health policy--lessons from the global burden of disease study. Science.

[3] Gorman JM (1996). Comorbid depression and anxiety spectrum disorders. Depress Anxiety.

[4] Samuels BA, Leonardo ED, Gadient R, Williams A, Zhou J, David DJ (2011). Modeling treatment-resistant depression. Neuropharmacology.

[5] Loomer HP, Saunders JC, Kline NS (1957). A clinical and pharmacodynamic evaluation of iproniazid as a psychic energizer. Psychiatr Res Rep Am Psychiatr Assoc.

[6] Tatsumi M, Groshan K, Blakely RD, Richelson E (1997). Pharmacological profile of antidepressants and related compounds at human monoamine transporters. Eur J Pharmacol.

[7] Glowinski J, Axelrod J (1964). Inhibition of uptake of Tritiated-Noradrenaline in the intact rat brain by Imipramine and structurally related compounds. Nature.

[8] Ross SB, Renyi AL (1969). Inhibition of the uptake of tritiated 5-hydroxytryptamine in brain tissue. Eur J Pharmacol.

[9] Feighner JP, Boyer WF (1996). Selective serotonin reuptake inhibitors, 2nd edition: advances in basic research and clinical practice.

[10] Stahl SM (1998). Mechanism of action of serotonin selective reuptake inhibitors. Serotonin receptors and pathways mediate therapeutic effects and side effects. J Affect Disord.

[11] Cohen E. CDC: Antidepressants most prescribed drugs in U.S.: CNN; 2007 [http://www.cnn.com/2007/HEALTH/07/09/antidepressants/]. Accessed 1 May 2017.

[12] Massart R, Mongeau R, Lanfumey L (2012). Beyond the monoaminergic hypothesis: neuroplasticity and epigenetic changes in a transgenic mouse model of depression. Philos Trans R Soc Lond B Biol Sci.

[13] Slattery DA, Hudson AL, Nutt DJ (2004). Invited review: the evolution of antidepressant mechanisms. Fundam Clin Pharmacol.

[14] Akiskal HS, McKinney WT (1973). Depressive disorders: toward a unified hypothesis. Science.

[15] Baumeister AA, Hawkins MF, Uzelac SM (2003). The myth of reserpine-induced depression: role in the historical development of the monoamine hypothesis. J Hist Neurosci.

[16] Leyton M, Young SN, Benkelfat C (1997). Relapse of depression after rapid depletion of tryptophan. Lancet.

[17] Delgado PL (2006). Monoamine depletion studies: implications for antidepressant discontinuation syndrome. J Clin Psychiatry.

[18] Jacobsen JP, Medvedev IO, Caron MG (2012). The 5-HT deficiency theory of depression: perspectives from a naturalistic 5-HT deficiency model, the tryptophan hydroxylase 2Arg439His knockin mouse. Philos Trans R Soc Lond B Biol Sci.

[19] Traskman L, Asberg M, Bertilsson L, Sjostrand L (1981). Monoamine metabolites in CSF and suicidal behavior. Arch Gen Psychiatry.

[20] Placidi GP, Oquendo MA, Malone KM, Huang YY, Ellis SP, Mann JJ (2001). Aggressivity, suicide attempts, and depression: relationship to cerebrospinal fluid monoamine metabolite levels. Biol Psychiatry.

[21] Asberg M (1997). Neurotransmitters and suicidal behavior. The evidence from cerebrospinal fluid studies. Ann N Y Acad Sci.

[22] Rush AJ, Trivedi MH, Wisniewski SR, Nierenberg AA, Stewart JW, Warden D (2006). Acute and longer-term outcomes in depressed outpatients requiring one or several treatment steps: a STAR*D report. Am J Psychiatry.

[23] Warden D, Rush AJ, Trivedi MH, Fava M, Wisniewski SR (2007). The STAR*D project results: a comprehensive review of findings. Curr Psychiatry Rep.

[24] Zarate CA, Singh JB, Carlson PJ, Brutsche NE, Ameli R, Luckenbaugh DA (2006). A randomized trial of an N-methyl-D-aspartate antagonist in treatment-resistant major depression. Arch Gen Psychiatry.

[25] Berman RM, Cappiello A, Anand A, Oren DA, Heninger GR, Charney DS (2000). Antidepressant effects of ketamine in depressed patients. Biol Psychiatry.

[26] Furey ML, Drevets WC (2006). Antidepressant efficacy of the antimuscarinic drug scopolamine: a randomized, placebo-controlled clinical trial. Arch Gen Psychiatry.

[27] Carlezon WA, Beguin C, Knoll AT, Cohen BM (2009). Kappa-opioid ligands in the study and treatment of mood disorders. Pharmacol Ther.

[28] Lutz PE, Kieffer BL (2013). Opioid receptors: distinct roles in mood disorders. Trends Neurosci.

[29] Samuels BA, Nautiyal KM, Kruegel AC, Levinstein MR, Magalong VM, Gassaway MM, et al. The Behavioral Effects of the Antidepressant Tianeptine Require the Mu-Opioid Receptor. Neuropsychopharmacology. 2017. Epub ahead of print. 10.1038/npp.2017.60 PMC5561344 28303899

[30] Caspi A, Sugden K, Moffitt TE, Taylor A, Craig IW, Harrington H (2003). Influence of life stress on depression: moderation by a polymorphism in the 5-HTT gene. Science.

[31] Lesch KP, Bengel D, Heils A, Sabol SZ, Greenberg BD, Petri S (1996). Association of anxiety-related traits with a polymorphism in the serotonin transporter gene regulatory region. Science.

[32] Bosker FJ, Hartman CA, Nolte IM, Prins BP, Terpstra P, Posthuma D (2011). Poor replication of candidate genes for major depressive disorder using genome-wide association data. Mol Psychiatry.

[33] Holmes A, Murphy DL, Crawley JN (2003). Abnormal behavioral phenotypes of serotonin transporter knockout mice: parallels with human anxiety and depression. Biol Psychiatry.

[34] Lira A, Zhou M, Castanon N, Ansorge MS, Gordon JA, Francis JH (2003). Altered depression-related behaviors and functional changes in the dorsal raphe nucleus of serotonin transporter-deficient mice. Biol Psychiatry.

[35] Beck SG, Choi KC, List TJ (1992). Comparison of 5-hydroxytryptamine1A-mediated hyperpolarization in CA1 and CA3 hippocampal pyramidal cells. J Pharmacol Exp Ther.

[36] Hamon M, Lanfumey L, el Mestikawy S, Boni C, Miquel MC, Bolanos F (1990). The main features of central 5-HT1 receptors. Neuropsychopharmacology.

[37] Riad M, Garcia S, Watkins KC, Jodoin N, Doucet E, Langlois X (2000). Somatodendritic localization of 5-HT1A and preterminal axonal localization of 5-HT1B serotonin receptors in adult rat brain. J Comp Neurol.

[38] Richardson-Jones JW, Craige CP, Guiard BP, Stephen A, Metzger KL, Kung HF (2010). 5-HT1A autoreceptor levels determine vulnerability to stress and response to antidepressants. Neuron.

[39] Hannon J, Hoyer D (2008). Molecular biology of 5-HT receptors. Behav Brain Res.

[40] Blier P, Pineyro G, el Mansari M, Bergeron R, de Montigny C (1998). Role of somatodendritic 5-HT autoreceptors in modulating 5-HT neurotransmission. Ann N Y Acad Sci.

[41] Richardson-Jones JW, Craige CP, Nguyen TH, Kung HF, Gardier AM, Dranovsky A (2011). Serotonin-1A autoreceptors are necessary and sufficient for the normal formation of circuits underlying innate anxiety. J Neurosci.

[42] Gross C, Zhuang X, Stark K, Ramboz S, Oosting R, Kirby L (2002). Serotonin1A receptor acts during development to establish normal anxiety-like behaviour in the adult. Nature.

[43] Ramboz S, Oosting R, Amara DA, Kung HF, Blier P, Mendelsohn M (1998). Serotonin receptor 1A knockout: an animal model of anxiety-related disorder. Proc Natl Acad Sci U S A.

[44] Wu S, Comings DE (1999). A common C-1018G polymorphism in the human 5-HT1A receptor gene. Psychiatr Genet.

[45] Lemonde S, Turecki G, Bakish D, Du L, Hrdina PD, Bown CD (2003). Impaired repression at a 5-hydroxytryptamine 1A receptor gene polymorphism associated with major depression and suicide. J Neurosci.

[46] Albert PR, Lemonde S (2004). 5-HT1A receptors, gene repression, and depression: guilt by association. Neuroscientist.

[47] Czesak M, Le Francois B, Millar AM, Deria M, Daigle M, Visvader JE (2012). Increased serotonin-1A (5-HT1A) autoreceptor expression and reduced raphe serotonin levels in deformed epidermal autoregulatory factor-1 (Deaf-1) gene knock-out mice. J Biol Chem.

[48] Parsey RV, Hastings RS, Oquendo MA, Huang YY, Simpson N, Arcement J (2006). Lower serotonin transporter binding potential in the human brain during major depressive episodes. Am J Psychiatry.

[49] Parsey RV, Oquendo MA, Ogden RT, Olvet DM, Simpson N, Huang YY (2006). Altered serotonin 1A binding in major depression: a [carbonyl-C-11]WAY100635 positron emission tomography study. Biol Psychiatry.

[50] Hamon M, Blier P (2013). Monoamine neurocircuitry in depression and strategies for new treatments. Prog Neuro-Psychopharmacol Biol Psychiatry.

[51] Lanfumey L, Hamon M (2004). 5-HT1 receptors. Curr Drug Targets CNS Neurol Disord.

[52] Samuels BA, Hen R (2011). Novelty-suppressed feeding in the mouse. In: Gould TD, editor. Mood and anxiety related phenotypes in mice: characterization using behavioral tests, volume II. Neuromethods.

[53] Garcia-Garcia AL, Newman-Tancredi A, Leonardo ED (2014). 5-HT(1A) [corrected] receptors in mood and anxiety: recent insights into autoreceptor versus heteroreceptor function. Psychopharmacology.

[54] Samuels BA, Anacker C, Hu A, Levinstein MR, Pickenhagen A, Tsetsenis T (2015). 5-HT1A receptors on mature dentate gyrus granule cells are critical for the antidepressant response. Nat Neurosci.

[55] Tanaka KF, Samuels BA, Hen R (2012). Serotonin receptor expression along the dorsal-ventral axis of mouse hippocampus. Philos Trans R Soc Lond B Biol Sci.

[56] Santarelli L, Saxe M, Gross C, Surget A, Battaglia F, Dulawa S (2003). Requirement of hippocampal neurogenesis for the behavioral effects of antidepressants. Science.

[57] Malberg JE, Eisch AJ, Nestler EJ, Duman RS (2000). Chronic antidepressant treatment increases neurogenesis in adult rat hippocampus. J Neurosci.

[58] McAskill R, Mir S, Taylor D (1998). Pindolol augmentation of antidepressant therapy. Br J Psychiatry.

[59] Newman-Tancredi A, Kleven MS (2011). Comparative pharmacology of antipsychotics possessing combined dopamine D2 and serotonin 5-HT1A receptor properties. Psychopharmacology.

[60] Neumaier JF, Edwards E, Plotsky PM (2002). 5-HT(1B) mrna regulation in two animal models of altered stress reactivity. Biol Psychiatry.

[61] Ruf BM, Bhagwagar Z (2009). The 5-HT1B receptor: a novel target for the pathophysiology of depression. Curr Drug Targets.

[62] Chenu F, David DJ, Leroux-Nicollet I, Le Maitre E, Gardier AM, Bourin M (2008). Serotonin1B heteroreceptor activation induces an antidepressant-like effect in mice with an alteration of the serotonergic system. J Psychiatry Neurosci.

[63] Ding S, Li L, Zhou FM (2015). Robust presynaptic serotonin 5-HT(1B) receptor inhibition of the striatonigral output and its sensitization by chronic fluoxetine treatment. J Neurophysiol.

[64] Gardier AM, Trillat AC, Malagie I, David D, Hascoet M, Colombel MC (2001). 5-HT1B serotonin receptors and antidepressant effects of selective serotonin reuptake inhibitors. C R Acad Sci III.

[65] Liu Y, Kelly MA, Sexton TJ, Neumaier JF (2015). 5-HT1B autoreceptors differentially modulate the expression of conditioned fear in a circuit-specific manner. Neuroscience.

[66] Nautiyal KM, Tritschler L, Ahmari SE, David DJ, Gardier AM, Hen R (2016). A lack of serotonin 1B Autoreceptors results in decreased anxiety and depression-related behaviors. Neuropsychopharmacology.

[67] McDevitt RA, Hiroi R, Mackenzie SM, Robin NC, Cohn A, Kim JJ (2011). Serotonin 1B autoreceptors originating in the caudal dorsal raphe nucleus reduce expression of fear and depression-like behavior. Biol Psychiatry.

[68] Neumaier JF, Petty F, Kramer GL, Szot P, Hamblin MW (1997). Learned helplessness increases 5-hydroxytryptamine1B receptor mRNA levels in the rat dorsal raphe nucleus. Biol Psychiatry.

[69] Dawson LA, Hughes ZA, Starr KR, Storey JD, Bettelini L, Bacchi F (2006). Characterisation of the selective 5-HT1B receptor antagonist SB-616234-a (1-[6-(cis-3,5-dimethylpiperazin-1-yl)-2,3-dihydro-5-methoxyindol-1-yl]-1-[2′-met hyl-4′-(5-methyl-1,2,4-oxadiazol-3-yl)biphenyl-4-yl]methanone hydrochloride): in vivo neurochemical and behavioural evidence of anxiolytic/antidepressant activity. Neuropharmacology.

[70] Tatarczynska E, Klodzinska A, Chojnacka-Wojcik E (2002). Effects of combined administration of 5-HT1A and/or 5-HT1B receptor antagonists and paroxetine or fluoxetine in the forced swimming test in rats. Pol J Pharmacol.

[71] Tatarczynska E, Klodzinska A, Stachowicz K, Chojnacka-Wojcik E (2004). Effects of a selective 5-HT1B receptor agonist and antagonists in animal models of anxiety and depression. Behav Pharmacol.

[72] Tatarczynska E, Klodzinska A, Stachowicz K, Chojnacka-Wojcik E (2004). Effect of combined administration of 5-HT1A or 5-HT1B/1D receptor antagonists and antidepressants in the forced swimming test. Eur J Pharmacol.

[73] Banasr M, Hery M, Printemps R, Daszuta A (2004). Serotonin-induced increases in adult cell proliferation and neurogenesis are mediated through different and common 5-HT receptor subtypes in the dentate gyrus and the subventricular zone. Neuropsychopharmacology.

[74] Carr GV, Lucki I (2011). The role of serotonin receptor subtypes in treating depression: a review of animal studies. Psychopharmacology.

[75] Niswender CM, Herrick-Davis K, Dilley GE, Meltzer HY, Overholser JC, Stockmeier CA (2001). RNA editing of the human serotonin 5-HT2C receptor. Alterations in suicide and implications for serotonergic pharmacotherapy. Neuropsychopharmacology.

[76] Kennedy SH, Emsley R (2006). Placebo-controlled trial of agomelatine in the treatment of major depressive disorder. Eur Neuropsychopharmacol.

[77] Olie JP, Kasper S (2007). Efficacy of agomelatine, a MT1/MT2 receptor agonist with 5-HT2C antagonistic properties, in major depressive disorder. Int J Neuropsychopharmacol.

[78] Loo H, Hale A, D'Haenen H (2002). Determination of the dose of agomelatine, a melatoninergic agonist and selective 5-HT(2C) antagonist, in the treatment of major depressive disorder: a placebo-controlled dose range study. Int Clin Psychopharmacol.

[79] Rainer Q, Xia L, Guilloux JP, Gabriel C, Mocaer E, Hen R (2012). Beneficial behavioural and neurogenic effects of agomelatine in a model of depression/anxiety. Int J Neuropsychopharmacol.

[80] Papp M, Gruca P, Boyer PA, Mocaer E (2003). Effect of agomelatine in the chronic mild stress model of depression in the rat. Neuropsychopharmacology.

[81] Gorman JM, Liebowitz MR, Fyer AJ, Goetz D, Campeas RB, Fyer MR (1987). An open trial of fluoxetine in the treatment of panic attacks. J Clin Psychopharmacol.

[82] Westenberg HG, den Boer JA (1989). Serotonin-influencing drugs in the treatment of panic disorder. Psychopathology.

[83] Burghardt NS, Bush DE, McEwen BS, LeDoux JE (2007). Acute selective serotonin reuptake inhibitors increase conditioned fear expression: blockade with a 5-HT(2C) receptor antagonist. Biol Psychiatry.

[84] Dekeyne A, Denorme B, Monneyron S, Millan MJ (2000). Citalopram reduces social interaction in rats by activation of serotonin (5-HT)(2C) receptors. Neuropharmacology.

[85] Belzung C, Le Guisquet AM, Barreau S, Calatayud F (2001). An investigation of the mechanisms responsible for acute fluoxetine-induced anxiogenic-like effects in mice. Behav Pharmacol.

[86] Marcinkiewcz CA, Mazzone CM, D'Agostino G, Halladay LR, Hardaway JA, DiBerto JF (2016). Serotonin engages an anxiety and fear-promoting circuit in the extended amygdala. Nature.

[87] Ohtsuki T, Ishiguro H, Detera-Wadleigh SD, Toyota T, Shimizu H, Yamada K (2002). Association between serotonin 4 receptor gene polymorphisms and bipolar disorder in Japanese case-control samples and the NIMH genetics initiative bipolar pedigrees. Mol Psychiatry.

[88] Rosel P, Arranz B, Urretavizcaya M, Oros M, San L, Navarro MA (2004). Altered 5-HT2A and 5-HT4 postsynaptic receptors and their intracellular signalling systems IP3 and cAMP in brains from depressed violent suicide victims. Neuropsychobiology.

[89] Madsen K, Torstensen E, Holst KK, Haahr ME, Knorr U, Frokjaer VG, et al. Familial risk for major depression is associated with lower striatal 5-HT(4) receptor binding. Int J Neuropsychopharmacol. 2014;18(1):1-7. 10.1093/ijnp/pyu034 PMC4368872 25522384

[90] Compan V, Zhou M, Grailhe R, Gazzara RA, Martin R, Gingrich J (2004). Attenuated response to stress and novelty and hypersensitivity to seizures in 5-HT4 receptor knock-out mice. J Neurosci.

[91] Conductier G, Dusticier N, Lucas G, Cote F, Debonnel G, Daszuta A (2006). Adaptive changes in serotonin neurons of the raphe nuclei in 5-HT(4) receptor knock-out mouse. Eur J Neurosci.

[92] Lucas G, Rymar VV, Du J, Mnie-Filali O, Bisgaard C, Manta S (2007). Serotonin(4) (5-HT(4)) receptor agonists are putative antidepressants with a rapid onset of action. Neuron.

[93] Mendez-David I, David DJ, Darcet F, Wu MV, Kerdine-Romer S, Gardier AM (2014). Rapid anxiolytic effects of a 5-HT(4) receptor agonist are mediated by a neurogenesis-independent mechanism. Neuropsychopharmacology.

[94] Cryan JF, Lucki I (2000). 5-HT4 receptors do not mediate the antidepressant-like behavioral effects of fluoxetine in a modified forced swim test. Eur J Pharmacol.

[95] Carr GV, Schechter LE, Lucki I (2011). Antidepressant and anxiolytic effects of selective 5-HT6 receptor agonists in rats. Psychopharmacology.

[96] Wesolowska A (2010). Potential role of the 5-HT6 receptor in depression and anxiety: an overview of preclinical data. Pharmacol Rep.

[97] Wesolowska A, Nikiforuk A (2007). Effects of the brain-penetrant and selective 5-HT6 receptor antagonist SB-399885 in animal models of anxiety and depression. Neuropharmacology.

[98] Wesolowska A, Nikiforuk A (2008). The selective 5-HT(6) receptor antagonist SB-399885 enhances anti-immobility action of antidepressants in rats. Eur J Pharmacol.

[99] Svenningsson P, Tzavara ET, Qi H, Carruthers R, Witkin JM, Nomikos GG (2007). Biochemical and behavioral evidence for antidepressant-like effects of 5-HT6 receptor stimulation. J Neurosci.

[100] Laplante P, Diorio J, Meaney MJ (2002). Serotonin regulates hippocampal glucocorticoid receptor expression via a 5-HT7 receptor. Brain Res Dev Brain Res.

[101] Yau JL, Noble J, Seckl JR (2001). Acute restraint stress increases 5-HT7 receptor mRNA expression in the rat hippocampus. Neurosci Lett.

[102] Mullins UL, Gianutsos G, Eison AS (1999). Effects of antidepressants on 5-HT7 receptor regulation in the rat hypothalamus. Neuropsychopharmacology.

[103] Hedlund PB, Huitron-Resendiz S, Henriksen SJ, Sutcliffe JG (2005). 5-HT7 receptor inhibition and inactivation induce antidepressantlike behavior and sleep pattern. Biol Psychiatry.

[104] Bonaventure P, Kelly L, Aluisio L, Shelton J, Lord B, Galici R (2007). Selective blockade of 5-hydroxytryptamine (5-HT)7 receptors enhances 5-HT transmission, antidepressant-like behavior, and rapid eye movement sleep suppression induced by citalopram in rodents. J Pharmacol Exp Ther.

[105] Guscott M, Bristow LJ, Hadingham K, Rosahl TW, Beer MS, Stanton JA (2005). Genetic knockout and pharmacological blockade studies of the 5-HT7 receptor suggest therapeutic potential in depression. Neuropharmacology.

[106] Wesolowska A, Nikiforuk A, Stachowicz K (2006). Potential anxiolytic and antidepressant effects of the selective 5-HT7 receptor antagonist SB 269970 after intrahippocampal administration to rats. Eur J Pharmacol.

[107] Wesolowska A, Nikiforuk A, Stachowicz K, Tatarczynska E (2006). Effect of the selective 5-HT7 receptor antagonist SB 269970 in animal models of anxiety and depression. Neuropharmacology.

[108] Abbas AI, Hedlund PB, Huang XP, Tran TB, Meltzer HY, Roth BL (2009). Amisulpride is a potent 5-HT7 antagonist: relevance for antidepressant actions in vivo. Psychopharmacology.

[109] Castren E (2004). Neurotrophins as mediators of drug effects on mood, addiction, and neuroprotection. Mol Neurobiol.

[110] Duman RS, Li N (2012). A neurotrophic hypothesis of depression: role of synaptogenesis in the actions of NMDA receptor antagonists. Philos Trans R Soc Lond B Biol Sci.

[111] Duman RS, Monteggia LM (2006). A neurotrophic model for stress-related mood disorders. Biol Psychiatry.

[112] Jacobs BL, van Praag H, Gage FH (2000). Adult brain neurogenesis and psychiatry: a novel theory of depression. Mol Psychiatry.

[113] Miller BR, Hen R (2015). The current state of the neurogenic theory of depression and anxiety. Curr Opin Neurobiol.

[114] Fournier NM, Duman RS (2012). Role of vascular endothelial growth factor in adult hippocampal neurogenesis: implications for the pathophysiology and treatment of depression. Behav Brain Res.

[115] Krishnan V, Nestler EJ (2008). The molecular neurobiology of depression. Nature.

[116] Monteggia LM, Luikart B, Barrot M, Theobold D, Malkovska I, Nef S (2007). Brain-derived neurotrophic factor conditional knockouts show gender differences in depression-related behaviors. Biol Psychiatry.

[117] Karege F, Vaudan G, Schwald M, Perroud N, La Harpe R (2005). Neurotrophin levels in postmortem brains of suicide victims and the effects of antemortem diagnosis and psychotropic drugs. Brain Res Mol Brain Res.

[118] Chen B, Dowlatshahi D, MacQueen GM, Wang JF, Young LT (2001). Increased hippocampal BDNF immunoreactivity in subjects treated with antidepressant medication. Biol Psychiatry.

[119] Nibuya M, Morinobu S, Duman RS (1995). Regulation of BDNF and trkB mRNA in rat brain by chronic electroconvulsive seizure and antidepressant drug treatments. J Neurosci.

[120] Nestler EJ, Barrot M, DiLeone RJ, Eisch AJ, Gold SJ, Monteggia LM (2002). Neurobiology of depression. Neuron.

[121] Li Y, Luikart BW, Birnbaum S, Chen J, Kwon CH, Kernie SG (2008). TrkB regulates hippocampal neurogenesis and governs sensitivity to antidepressive treatment. Neuron.

[122] Sairanen M, Lucas G, Ernfors P, Castren M, Castren E (2005). Brain-derived neurotrophic factor and antidepressant drugs have different but coordinated effects on neuronal turnover, proliferation, and survival in the adult dentate gyrus. J Neurosci.

[123] Monteggia LM, Barrot M, Powell CM, Berton O, Galanis V, Gemelli T (2004). Essential role of brain-derived neurotrophic factor in adult hippocampal function. Proc Natl Acad Sci U S A.

[124] Wang J-W, David DJ, Monckton JE, Battaglia F (2008). Hen Rà. Chronic fluoxetine stimulates maturation and synaptic plasticity of adult-born hippocampal granule cells. J Neurosci.

[125] Castren E, Voikar V, Rantamaki T (2007). Role of neurotrophic factors in depression. Curr Opin Pharmacol.

[126] Rantamaki T, Hendolin P, Kankaanpaa A, Mijatovic J, Piepponen P, Domenici E (2007). Pharmacologically diverse antidepressants rapidly activate brain-derived neurotrophic factor receptor TrkB and induce phospholipase-Cgamma signaling pathways in mouse brain. Neuropsychopharmacology.

[127] Warner-Schmidt JL, Duman RS (2007). VEGF is an essential mediator of the neurogenic and behavioral actions of antidepressants. Proc Natl Acad Sci U S A.

[128] Fujioka T, Fujioka A, Duman RS (2004). Activation of cAMP signaling facilitates the morphological maturation of newborn neurons in adult hippocampus. J Neurosci.

[129] Shirayama Y, Chen AC, Nakagawa S, Russell DS, Duman RS (2002). Brain-derived neurotrophic factor produces antidepressant effects in behavioral models of depression. J Neurosci.

[130] Dow AL, Russell DS, Duman RS (2005). Regulation of activin mRNA and Smad2 phosphorylation by antidepressant treatment in the rat brain: effects in behavioral models. J Neurosci.

[131] Borroto-Escuela DO, Romero-Fernandez W, Mudo G, Perez-Alea M, Ciruela F, Tarakanov AO (2012). Fibroblast growth factor receptor 1- 5-hydroxytryptamine 1A heteroreceptor complexes and their enhancement of hippocampal plasticity. Biol Psychiatry.

[132] Ganea K, Menke A, Schmidt MV, Lucae S, Rammes G, Liebl C (2012). Convergent animal and human evidence suggests the activin/inhibin pathway to be involved in antidepressant response. Transl Psychiatry.

[133] Greene J, Banasr M, Lee B, Warner-Schmidt J, Duman RS (2009). Vascular endothelial growth factor signaling is required for the behavioral actions of antidepressant treatment: pharmacological and cellular characterization. Neuropsychopharmacology.

[134] Hoshaw BA, Malberg JE, Lucki I (2005). Central administration of IGF-I and BDNF leads to long-lasting antidepressant-like effects. Brain Res.

[135] Turner CA, Gula EL, Taylor LP, Watson SJ, Akil H (2008). Antidepressant-like effects of intracerebroventricular FGF2 in rats. Brain Res.

[136] Warner-Schmidt JL, Duman RS (2008). VEGF as a potential target for therapeutic intervention in depression. Curr Opin Pharmacol.

[137] Siuciak JA, Lewis DR, Wiegand SJ, Lindsay RM (1997). Antidepressant-like effect of brain-derived neurotrophic factor (BDNF). Pharmacol Biochem Behav.

[138] Lang UE, Hellweg R, Kalus P, Bajbouj M, Lenzen KP, Sander T (2005). Association of a functional BDNF polymorphism and anxiety-related personality traits. Psychopharmacology.

[139] Sen S, Nesse RM, Stoltenberg SF, Li S, Gleiberman L, Chakravarti A (2003). A BDNF coding variant is associated with the NEO personality inventory domain neuroticism, a risk factor for depression. Neuropsychopharmacology.

[140] Verhagen M, van der Meij A, van Deurzen PA, Janzing JG, Arias-Vasquez A, Buitelaar JK (2010). Meta-analysis of the BDNF Val66Met polymorphism in major depressive disorder: effects of gender and ethnicity. Mol Psychiatry.

[141] Hwang JP, Tsai SJ, Hong CJ, Yang CH, Lirng JF, Yang YM (2006). The Val66Met polymorphism of the brain-derived neurotrophic-factor gene is associated with geriatric depression. Neurobiol Aging.

[142] Iga J, Ueno S, Yamauchi K, Numata S, Tayoshi-Shibuya S, Kinouchi S (2007). The Val66Met polymorphism of the brain-derived neurotrophic factor gene is associated with psychotic feature and suicidal behavior in Japanese major depressive patients. Am J Med Genet B Neuropsychiatr Genet.

[143] Chen ZY, Jing D, Bath KG, Ieraci A, Khan T, Siao CJ (2006). Genetic variant BDNF (Val66Met) polymorphism alters anxiety-related behavior. Science.

[144] Anttila S, Huuhka K, Huuhka M, Rontu R, Hurme M, Leinonen E (2007). Interaction between 5-HT1A and BDNF genotypes increases the risk of treatment-resistant depression. J Neural Transm (Vienna).

[145] Kishi T, Tsunoka T, Ikeda M, Kawashima K, Okochi T, Kitajima T (2009). Serotonin 1A receptor gene and major depressive disorder: an association study and meta-analysis. J Hum Genet.

[146] Kishi T, Yoshimura R, Fukuo Y, Okochi T, Matsunaga S, Umene-Nakano W (2013). The serotonin 1A receptor gene confer susceptibility to mood disorders: results from an extended meta-analysis of patients with major depression and bipolar disorder. Eur Arch Psychiatry Clin Neurosci.

[147] Autry AE, Monteggia LM (2012). Brain-derived neurotrophic factor and neuropsychiatric disorders. Pharmacol Rev.

[148] Martinowich K, Lu B (2008). Interaction between BDNF and serotonin: role in mood disorders. Neuropsychopharmacology.

[149] Gould E (1999). Serotonin and hippocampal neurogenesis. Neuropsychopharmacology.

[150] Mossner R, Daniel S, Albert D, Heils A, Okladnova O, Schmitt A (2000). Serotonin transporter function is modulated by brain-derived neurotrophic factor (BDNF) but not nerve growth factor (NGF). Neurochem Int.

[151] Pascual-Brazo J, Castro E, Diaz A, Valdizan EM, Pilar-Cuellar F, Vidal R (2012). Modulation of neuroplasticity pathways and antidepressant-like behavioural responses following the short-term (3 and 7 days) administration of the 5-HT(4) receptor agonist RS67333. Int J Neuropsychopharmacol.

[152] Samarajeewa A, Goldemann L, Vasefi MS, Ahmed N, Gondora N, Khanderia C (2014). 5-HT7 receptor activation promotes an increase in TrkB receptor expression and phosphorylation. Front Behav Neurosci.

[153] Cao L, Jiao X, Zuzga DS, Liu Y, Fong DM, Young D (2004). VEGF links hippocampal activity with neurogenesis, learning and memory. Nat Genet.

[154] Borroto-Escuela DO, Tarakanov AO, Fuxe K (2016). FGFR1-5-HT1A Heteroreceptor complexes: implications for understanding and treating major depression. Trends Neurosci.

[155] Borroto-Escuela DO, Narvaez M, Perez-Alea M, Tarakanov AO, Jimenez-Beristain A, Mudo G (2015). Evidence for the existence of FGFR1-5-HT1A heteroreceptor complexes in the midbrain raphe 5-HT system. Biochem Biophys Res Commun.

[156] Borroto-Escuela DO, Perez-Alea M, Narvaez M, Tarakanov AO, Mudo G, Jimenez-Beristain A (2015). Enhancement of the FGFR1 signaling in the FGFR1-5-HT1A heteroreceptor complex in midbrain raphe 5-HT neuron systems. Relevance for neuroplasticity and depression. Biochem Biophys Res Commun.

[157] Ming G-L, Song H (2005). Adult neurogenesis in the mammalian central nervous system. Annu Rev Neurosci.

[158] Suh H, Deng W, Gage F (2009). Signaling in Adult Neurogenesis. Annu Rev Cell Dev Biol.

[159] Malberg JE (2004). Implications of adult hippocampal neurogenesis in antidepressant action. J Psychiatry Neurosci.

[160] Airan RD, Meltzer LA, Roy M, Gong Y, Chen H, Deisseroth K (2007). High-speed imaging reveals neurophysiological links to behavior in an animal model of depression. Science.

[161] David DJ, Samuels BA, Rainer Q, Wang JW, Marsteller D, Mendez I (2009). Neurogenesis-dependent and -independent effects of fluoxetine in an animal model of anxiety/depression. Neuron.

[162] Boldrini M, Santiago AN, Hen R, Dwork AJ, Rosoklija GB, Tamir H (2013). Hippocampal granule neuron number and dentate gyrus volume in antidepressant-treated and untreated major depression. Neuropsychopharmacology.

[163] Sahay A, Scobie KN, Hill AS, O'Carroll CM, Kheirbek MA, Burghardt NS (2011). Increasing adult hippocampal neurogenesis is sufficient to improve pattern separation. Nature.

[164] Ishizuka T, Goshima H, Ozawa A, Watanabe Y (2014). Stimulation of 5-HT4 receptor enhances differentiation of mouse induced pluripotent stem cells into neural progenitor cells. Clin Exp Pharmacol Physiol.

[165] Tamburella A, Micale V, Navarria A, Drago F (2009). Antidepressant properties of the 5-HT4 receptor partial agonist, SL65.0155: behavioral and neurochemical studies in rats. Prog Neuro-Psychopharmacol Biol Psychiatry.

[166] Liu MT, Kuan YH, Wang J, Hen R, Gershon MD (2009). 5-HT4 receptor-mediated neuroprotection and neurogenesis in the enteric nervous system of adult mice. J Neurosci.

[167] Imoto Y, Kira T, Sukeno M, Nishitani N, Nagayasu K, Nakagawa T (2015). Role of the 5-HT4 receptor in chronic fluoxetine treatment-induced neurogenic activity and granule cell dematuration in the dentate gyrus. Mol Brain..

[168] Kobayashi K, Ikeda Y, Sakai A, Yamasaki N, Haneda E, Miyakawa T (2010). Reversal of hippocampal neuronal maturation by serotonergic antidepressants. Proc Natl Acad Sci U S A.

[169] Karpova NN, Pickenhagen A, Lindholm J, Tiraboschi E, Kulesskaya N, Agustsdottir A (2011). Fear erasure in mice requires synergy between antidepressant drugs and extinction training. Science.

[170] Ohira K, Takeuchi R, Iwanaga T, Miyakawa T (2013). Chronic fluoxetine treatment reduces parvalbumin expression and perineuronal nets in gamma-aminobutyric acidergic interneurons of the frontal cortex in adult mice. Mol Brain.

[171] Lee DA, Blackshaw S (2012). Functional implications of hypothalamic neurogenesis in the adult mammalian brain. Int J Dev Neurosci.

[172] Gould E (2007). How widespread is adult neurogenesis in mammals?. Nat Rev Neurosci.

[173] Ohira K, Takeuchi R, Shoji H, Miyakawa T (2013). Fluoxetine-induced cortical adult neurogenesis. Neuropsychopharmacology.

[174] Luscher B, Shen Q, Sahir N (2011). The GABAergic deficit hypothesis of major depressive disorder. Mol Psychiatry.

[175] Burghardt NS, Park EH, Hen R, Fenton AA (2012). Adult-born hippocampal neurons promote cognitive flexibility in mice. Hippocampus.

[176] Drew LJ, Kheirbek MA, Luna VM, Denny CA, Cloidt MA, Wu MV (2016). Activation of local inhibitory circuits in the dentate gyrus by adult-born neurons. Hippocampus.

[177] Ikrar T, Guo N, He K, Besnard A, Levinson S, Hill A (2013). Adult neurogenesis modifies excitability of the dentate gyrus. Front Neural Circuits.

[178] Lacefield CO, Itskov V, Reardon T, Hen R, Gordon JA (2012). Effects of adult-generated granule cells on coordinated network activity in the dentate gyrus. Hippocampus.

[179] Fanselow MS, Dong HW (2010). Are the dorsal and ventral hippocampus functionally distinct structures?. Neuron.

[180] Pothuizen HH, Zhang WN, Jongen-Relo AL, Feldon J, Yee BK (2004). Dissociation of function between the dorsal and the ventral hippocampus in spatial learning abilities of the rat: a within-subject, within-task comparison of reference and working spatial memory. Eur J Neurosci.

[181] Kheirbek MA, Drew LJ, Burghardt NS, Costantini DO, Tannenholz L, Ahmari SE (2013). Differential control of learning and anxiety along the dorsoventral axis of the dentate gyrus. Neuron.

[182] Bagot RC, Parise EM, Pena CJ, Zhang HX, Maze I, Chaudhury D (2015). Ventral hippocampal afferents to the nucleus accumbens regulate susceptibility to depression. Nat Commun.

[183] Tye KM, Prakash R, Kim SY, Fenno LE, Grosenick L, Zarabi H (2011). Amygdala circuitry mediating reversible and bidirectional control of anxiety. Nature.

[184] Padilla-Coreano N, Bolkan SS, Pierce GM, Blackman DR, Hardin WD, Garcia-Garcia AL, et al. Direct ventral hippocampal-prefrontal input is required for anxiety-related neural activity and behavior. Neuron. 2016;89(4):857–66. 10.1016/j.neuron.2016.01.011 PMC4760847 26853301

[185] Wu MV, Sahay A, Duman RS, Hen R (2015). Functional differentiation of adult-born neurons along the septotemporal axis of the dentate gyrus. Cold Spring Harb Perspect Biol.

[186] Gage FH, Thompson RG, Valdes JJ (1978). Endogenous norepinephrine and serotonin within the hippocampal formation during the development and recovery from septal hyperreactivity. Pharmacol Biochem Behav.

